# Identification of Discharge Pathways of Acidic Wastewater from a Bauxite Mine (Lujiang Alum Mine, China) Before and After Artificial Disturbance

**DOI:** 10.3390/toxics14010051

**Published:** 2025-12-31

**Authors:** Wenming Wang, Weichao Jia, Lin Xu, Zhenyu He, Bo Kang, Kun Chen

**Affiliations:** 1 School of Environmental Science and Engineering, Tianjin University, Tianjin 300072, China; wangwenming1980@163.com; 2 PowerChina Northwest Engineering Corporation Limited, Xi’an 710100, China; jiaweichao@nwh.cn (W.J.); fly20231025@163.com (L.X.); 3 School of Civil Engineering, Hefei University of Technology, Hefei 230009, China; 4 School of Resources and Environmental Engineering, Hefei University of Technology, Hefei 230009, China; 5 Anhui Survey and Design Institute of Water Conservancy and Hydropower Co., Ltd., Hefei 230009, China

**Keywords:** acid mine wastewater, disturbance remediation, environmental tracing, water source variation

## Abstract

This study focuses on the Lujiang Alum Mine, analyzing sources of acid mine drainage (AMD) generated during remediation activities. A numerical model of groundwater flow was constructed to simulate and predict the causes of AMD under the influence of remediation measures. Concurrently, hydrogen and oxygen stable-isotope-tracing techniques were employed to elucidate the pathways through which AMD occurred and the mechanisms underlying water acidification. A fully mixed model was established to quantify the rates of contribution from different water sources. The results indicate that the annual amount of acidic wastewater produced under the influence of disturbance via remediation is approximately 3.29 × 10^5^ m^3^. The fully mixed model based on environmental isotopes further revealed that the discharge of water from the first branch of the +85 m adit serves as the primary cause of AMD during the wet, normal, and dry seasons, with a contribution exceeding 50%. This is followed by recharge from Tianchi Lake, accounting for approximately 20–30%. In contrast, the contributions from seepage water from the roof of the +85 m adit and water from the Xiaofanshan Inclined Shaft are relatively minor. Based on these findings, we propose targeted strategies for source prevention and end-of-pipe treatment of AMD in the mining area. This work provides scientific support for the ongoing ecological restoration project at the Lujiang Alum Mine and offers valuable insights for AMD management in similar mines.

## 1. Introduction

China, which possesses substantial mineral reserves and a robust mining industry, plays a pivotal role in national socioeconomic development. Nevertheless, mining activities represent one of the most extensive forms of human-induced alteration of terrestrial ecosystems and surface landscapes [1]. In the early phases of mineral exploitation, limited emphasis was placed on environmental protection and systematic management. Coupled with prolonged and frequently disordered extraction practices, this oversight resulted in numerous abandoned mining sites plagued by diverse ecological and environmental challenges [2,3,4]. China began work on the ecological restoration of mines relatively late, with underdeveloped technological frameworks and institutional mechanisms. Although notable advances have been made in recent years, persistent shortcomings are reflected in suboptimal engineering outcomes, representing a significant practical impediment [5].

Acid mine drainage (AMD) pollution is one of the major challenges in the ecological restoration of abandoned mines and represents a global environmental issue [6,7,8,9]. AMD wastewater is a sulfate-rich solution characterized by high concentrations of heavy metal ions, elevated sulfate levels, low pH, and a high heavy metal content [10,11,12]. These characteristics result from the reaction of precipitation—during runoff and infiltration—with residual tailings and metal sulfides (such as alumite (whose primary component is KAl(SO_4_)_2_·12H_2_O) and pyrite (whose chemical formula is FeS_2_)) left over after mining activities or present in unmined ore bodies. These reactions continuously release hydrogen ions, sulfate ions, and various heavy metal ions [13,14]. The occurrence of AMD is controlled by multiple factors (biological, physical, and chemical) and significantly influenced by environmental conditions. Therefore, clarifying the sources and formation mechanisms of acidic water is undoubtedly a cornerstone of ecological restoration of mines [15,16].

We employed the Lujiang Alum Mine, influenced by artificial restoration disturbance, as a case study, analyzing the mechanisms and sources of water acidification relative to pre-restoration conditions. Before restoration activities were conducted, the recharge patterns of acidic water primarily encompassed three modes: direct recharge at adit openings, seepage recharge through collapsed areas, and regional seepage flow. Surface remediation projects mainly involve the treatment of open-pit mining areas, old adit openings, collapse zones, and roadways. Groundwater remediation measures include in situ treatment of acidic mine water in pits and the use of ecological filtration beds. Most of these engineering measures have now been widely implemented. Through an analysis of hydrogeological data and field investigations conducted at the alum mine, the water–rock interactions during groundwater seepage were analyzed, elucidating the acidification mechanism of AMD in the study area. This research provides a theoretical basis for further promoting ecological restoration projects in alum mines, is of significant practical importance for the ecological protection of Chaohu Lake and the Yangtze River, and offers a valuable reference for AMD treatment and the ecological restoration of abandoned mines.

## 2. Materials and Methods

### 2.1. Sample Collection

**Figure 1:**
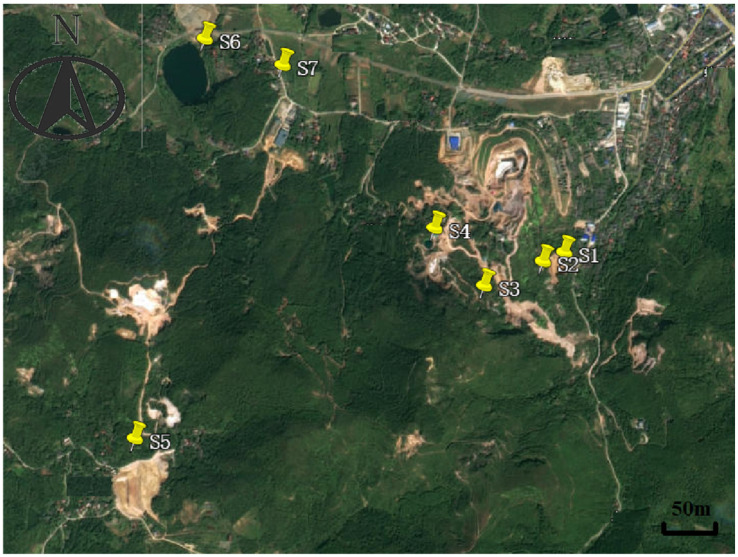
Sampling point distribution in this study.

This study involved the collection of water samples from multiple underground and surface locations at the alum mine, including adit discharge. The sampling frequency varied for different analytical purposes. The details of the sampling points are provided in Table 1, the corresponding testing frequencies are listed in Table 1 and Table 2, and their distribution is illustrated in Figure 1. The collection methods and requirements for various test components were implemented in accordance with the following protocols.

**Figure 2:**
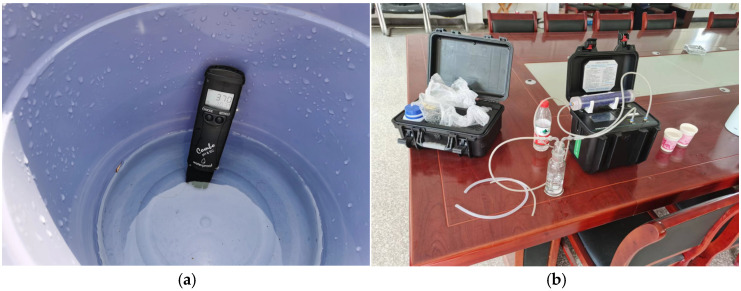
Photo of a sample during the test.

General Sampling Protocol: To collect surface water and adit discharge samples, we rinsed the sample container 2–3 times with the target water (a step applied to all containers for subsequent component analysis) before immersing it in the water to collect the sample. Care was taken to avoid disturbing bottom sediments during sampling to prevent impacts on analytical results. For groundwater sampling, with the exception of the difficult-to-access Xiaofanshan Inclined Shaft and vertical shafts, well pumping (or purging) was performed prior to sampling, typically displacing 3–5 times the standing water volume in the well column, to eliminate the influence of stagnant water on the test results. Before sample collection, a Hanna portable meter was used for on-site measurement of pH, EC (Electrical Conductivity), water temperature, and TDS (Total Dissolved Solids) (Figure 2). Sampling commenced only after these parameters stabilized. After filling the containers, we verified that their seals were intact. Furthermore, for research purposes, the flow rate of acidic wastewater from the +85 m adit portal was measured on-site using a handheld ultrasonic flowmeter.

Water Chemistry Samples: Water samples for chemical analysis were stored in polyethylene bottles. Prior to being filled, each sample bottle was rinsed 2–3 times with the water to be sampled. Each bottle was filled completely, ensuring no air bubbles remained; sealed with Parafilm; and stored in a cool, dark place.

Hydrogen (δ^2^H) and Oxygen (δ^18^O) Isotope Samples: Samples for hydrogen and oxygen isotope analysis were filtered through a 0.22 μm MCE membrane; placed into pre-rinsed 2 mL LABCO bottles, leaving a small amount of headspace; and then sealed and refrigerated for storage [17].

Radon (^222^Rn) Concentration Measurement: To avoid measurement bias caused by radon decay, radon concentrations were determined via on-site measurement. Before each measurement, the radon-sampling vial was rinsed with the water sample to be tested. Subsequently, 100 mL of the water sample was poured into the vial, and measurement was initiated immediately. ^222^Rn is a decay product of radium, with a half-life of approximately 3.825 days. It reaches radioactive equilibrium with its parent radium within about one month [18].

In this study, radon isotope testing of the samples was conducted using an environmental radon monitor (Instrument Model No. HS01B) manufactured by Chengdu Hesheng Technology Co., Ltd. (Chengdu, China). This instrument employs a high-resolution gold–silicon surface barrier semiconductor α-ray detector and is developed around a microcontroller as its core, representing a new generation of intelligent radiation protection detection equipment that measures radon via α-spectrometry.

Since there is no international benchmark for measuring radon in water—primarily because such measurements involve the amount of radon escaping from water, and there are no global standards for quantifying this outgassing ratio—radon-in-water measurements can only provide reference values relative to radon measurements in air. The HS01B instrument directs air samples through a fine-pored filter membrane (which removes radon progeny) into a radon collection chamber. Inside this chamber, radon gas decays and produces progeny. By measuring these progeny, the instrument effectively measures radon concentrations. This radon monitor is notable for its user-friendliness and high accuracy.

### 2.2. Hydrogeological Conceptual Model

#### 2.2.1. Aquifer Generalization

Based on regional hydrogeological data, the aquifer formations in the study area were generalized from top to bottom into four aquifer groups: a loose-rock porous aquifer group (characterized by pore water and shallow burial depth), a volcaniclastic rock pore-fissure aquifer group (composed mainly of trachyte porphyry and tuff, notable for its great thickness, wide distribution, and moderate abundance in water), a carbonate rock karst-fissure aquifer group (consisting of dolomitic sandstone and limestone from the Luoling Formation, with moderate water-yielding capacity), and a magmatic rock fissure aquifer (composed of syenite and quartz syenite, characterized by very low permeability and poor water yield).

According to stratigraphic boundaries, fracture development degrees, and the distribution of underground workings, this simulation primarily focuses on the strata above the +85 m adit. Consequently, the formations within the study area were generalized into five layers:

Layer 1: The first model layer primarily consists of weathered crust and backfill soil from remediation engineering. The weathered crust predominantly consists of the loose-rock porous aquifer group.

Layer 2: The fracture development zone between the bottom of Layer 1 and an elevation of +135 m was generalized as the second model layer. This stratum is primarily composed of members of the volcaniclastic rock fissure aquifer group.

Layer 3: The third model layer corresponds to the stratum where the +132 m adit is located. As the height of this adit is 3 m, this layer spans the elevation interval from +132 m to +135 m.

Layer 4: The fourth model layer comprises the strata between the bottom of the +132 m adit and the top of the +85 m adit, spanning elevations from +88 m to +135 m. This layer mainly consists of members of the volcaniclastic rock fissure aquifer group.

Layer 5: The fifth model layer is the stratum containing the +85 m adit. Given the adit’s height of 3 m, this layer covers the elevation range from +85 m to +88 m.

#### 2.2.2. Boundary Condition Generalization

The western and southern boundaries of the study area are defined by topographic divides, while the northern boundary is delineated by a groundwater equipotential line, and the eastern boundary is marked by the Fanshan normal fault. The total area of the study area is approximately 0.67 km^2^.

The western and southern boundaries, constrained by topography and characterized by higher elevations, were generalized as no-flow boundaries. Based on the analysis of groundwater recharge, flow, and discharge conditions, the northern boundary was also generalized as a no-flow boundary. The eastern boundary, defined by the Fanshan normal fault—a moderately water-bearing fracture zone—acts as a discharge boundary due to the general south-to-north groundwater flow direction and was thus generalized as a specified-flux boundary.

The upper boundary of the model primarily receives atmospheric precipitation recharge and is subject to phreatic water evaporation; therefore, it was generalized as a boundary for precipitation infiltration recharge and evaporation. Since the main drainage channel of the +85 m adit currently serves as the primary discharge outlet for acidic mine drainage, our numerical simulation of groundwater in the mining area focuses solely on acidic water discharge at the +85 m adit portal. Accordingly, the lower boundary of the model was generalized as a no-flow boundary.

#### 2.2.3. The Hydrogeological Parameters and Source/Sink Terms for the Model

Numerous subsidence zones, goafs, and mining adits have been developed in the underground area in the studied region due to mining activities. Although the ecological restoration project involved backfilling and land leveling in the subsidence areas and goafs, the hydrological and geological conditions in the restored subsidence zones and quarry openings still differ from those in the surrounding strata. Moreover, the zoning of hydrogeological parameters and the selection of values for each aquifer also vary. Based on existing mining data, restoration-engineering records, and geophysical survey data from the mining area, we conducted parameter zoning for the study area (Table 3).

The study area is primarily influenced by rainfall, with recharge sources including surface infiltration recharge, recharge through mining-waste subsidence areas, and direct “window-like” infiltration recharge.

### 2.3. Mathematical Model

The aquifer media can be conceptualized as heterogeneous and anisotropic continuous. The lithology of the aquifer varies significantly at different locations, and its hydrogeological parameters change spatially. Consequently, the aquifer under consideration can be represented as a heterogeneous, anisotropic, and quasi-three-dimensional groundwater flow system, for which a corresponding mathematical model can be established.

(1) $$\left\{\begin{array}{l} \frac{\partial }{\partial x}\left(K_{x}\frac{\partial h}{\partial x}\right)+\frac{\partial }{\partial y}\left(K_{y}\frac{\partial h}{\partial y}\right)+\frac{\partial }{\partial z}\left(K_{z}\frac{\partial h}{\partial z}\right)+W=\mu_{s}\frac{\partial h}{\partial t}\\ {\left.h\left(x,y,z,t\right)\right|}_{t=0}=h_{0}\left(x,y,z\right),\;\left(x,y,z\right)\in D\\ {\left.h\left(x,y,z,t\right)\right|}_{\Gamma_{1}}=h\left(x,y,z,t\right),\;\left(x,y,z\right)\in \Gamma_{1},t\geq 0\\ K\frac{\partial h}{\partial \vec{n}}{|}_{\Gamma_{2}}=q\left(x,y,z,t\right),\;\;\;\left(x,y,z\right)\in \Gamma_{2},t>0 \end{array}\right.$$

Here, *K_x_*, *K_y_*, and *K_z_* are components of the hydraulic conductivity tensor in the *x*, *y*, and *z* directions (m/d), respectively (it is assumed that the principal axes of the hydraulic conductivity tensor are aligned with the coordinate axes); *h* denotes groundwater head (m); *W* denotes volumetric flux per unit volume (1/d), representing sources (recharge) and sinks (discharge); *μ_s_* denotes specific storage of the aquifer system (1/m); *h*_0_(*x*,*y*,*z*) denotes known initial hydraulic head distribution (m); *t* denotes time (d); *D* denotes the domain of the simulation area; Γ_1_ denotes the specified-head (Dirichlet) boundary; Γ_2_ denotes the specified-flux (Neumann) boundary; and *q*(*x*,*y*,*z*,*t*) denotes known flux distribution across the specified-flux boundary.

### 2.4. Numerical Simulation Model

In this section, we used the MODFLOW, MODPATH, and MT3DMS modules in GMS 10.9 software to establish a three-dimensional model of the dynamic changes in groundwater for the area. By generalizing the hydrogeological conditions, boundary conditions, and source/sink terms of the study area, a mathematical model of acidic groundwater wastewater was developed. Based on the existing data from the study area, the trial-estimation correction method was applied to adjust the existing parameters. The model parameters were then calibrated and validated to enhance consistency between the model and the actual conditions, in turn ensuring that the simulated groundwater flow field aligned with the real flow field in the region.

**Figure 3:**
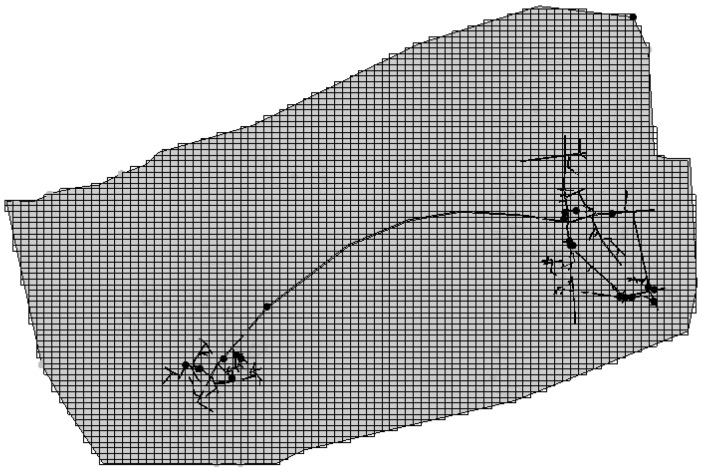
Simulated regional grid profile.

**Figure 4:**
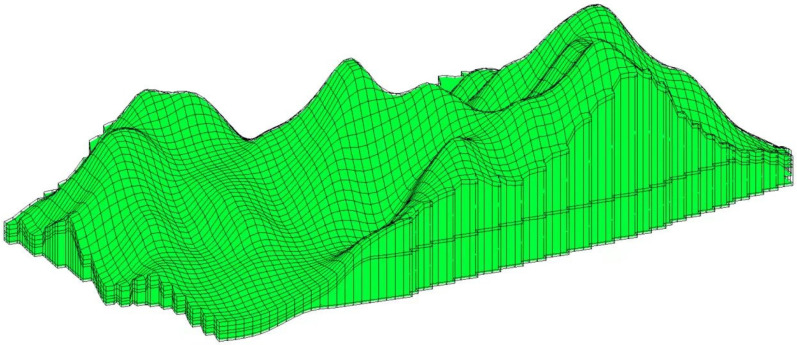
Three-dimensional schematic diagram of the model.

Based on the hydrogeological conceptual model of the study area, which incorporates rock fracture development, stratigraphic boundaries, and the distribution of underground mining adits as interpreted from regional geological cross-sections, the strata in the numerical simulation model were generalized into five distinct layers. The discretized model area was then divided in the two-dimensional plane into a structured grid consisting of 100 rows by 100 columns, resulting in a total of 10,000 cells. Following grid generation, cells located outside the actual simulation area were defined as inactive (no-flow boundaries). The resulting spatial discretization, including the layering scheme and the distribution of active versus inactive cells, is visually represented in the simulation domain grid layout provided in Figure 3 and Figure 4.

**Figure 5:**
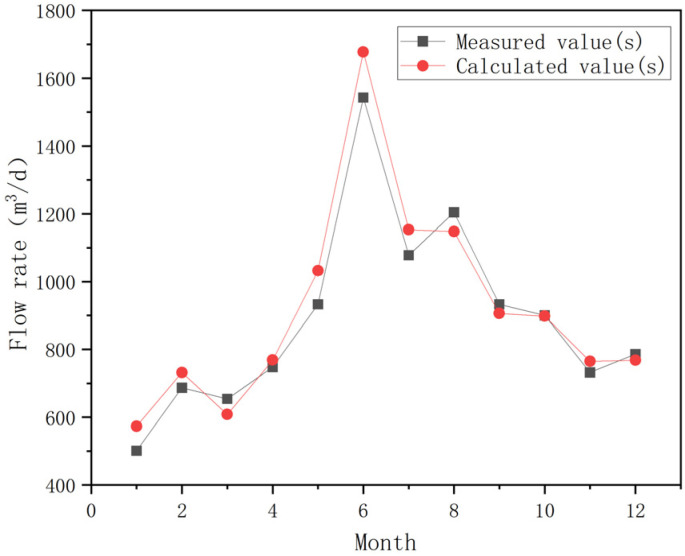
Verification results regarding acidic drainage discharge at the observation point during the verification period.

To enhance the model’s accuracy and constrain the uncertainties associated with acid-water-monitoring data and regional hydrogeological conditions, we further validated the calibrated model, as required. The validation period was set from January 2024 to December 2024, with January 2024 serving as the start time for this phase. The handling of source and sink terms remained consistent with the calibration period, and the parameter values finalized during calibration were adopted. The validation results are presented in Figure 5.

## 3. Results

### 3.1. Solute Transport in the Groundwater Flow System of the Mining Area

Based on the test results, we summarized the water quality data for the main drainage channel at the +85 m adit and the recharge sources under the four recharge scenarios in Table 4. The data presented in the table were obtained through the following steps: First, standardized sampling and pretreatment were carried out for water samples from each sampling point. The samples were then sent to the laboratory, where major ions and metal elements were analyzed according to standard methods. Finally, the test results were validated and organized to generate the comparative data shown in the table, ensuring the traceability and reliability of the data.

### 3.2. Prediction of Acidic Mine Water Discharge

**Figure 6:**
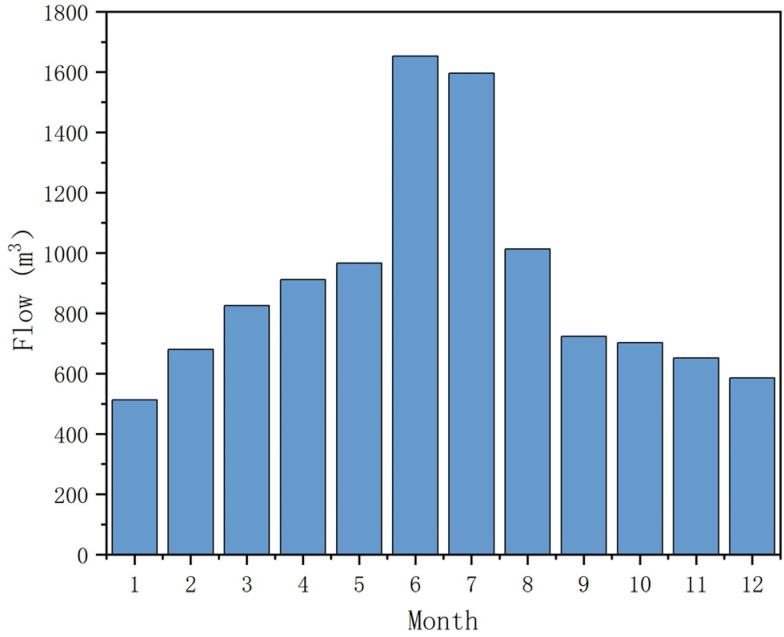
Predicted results from the acidic water output model after remediation.

Based on the established groundwater flow numerical model, we predicted the discharge rate of acidic mine water under the influence of multi-year average precipitation and evaporation conditions after the implementation of the ecological restoration project. The intra-annual dynamics and acidic water generation during the wet and dry seasons were analyzed. The specific prediction results are shown in Figure 6.

As illustrated in the figure, following the implementation of the restoration project, the total annual volume of acidic water generated was 3.29 × 10^5^ m^3^/a. The maximum daily discharge was 1653 m^3^/d, the minimum was 513 m^3^/d, and the average was 902 m^3^/d.

Lujiang County is located in the Jiang-Huai Hilly Region. For the delineation of wet and dry seasons in this area, one can refer to Fang G. et al.’s classification of precipitation stages in Hefei [19]. According to this classification, the wet season occurs in June, July, and August; the dry season is in December, January, and February; and the remaining months are considered the normal season.

The numerical model predictions indicate that the average discharge rate of acidic water during the wet season is 1421 m^3^/d, 797 m^3^/d during the normal season, and 593 m^3^/d during the dry season.

### 3.3. Proportional Contribution of Different Sources to Acidic Mine Water Discharge During Different Seasons

The contribution of various types of source water to the acidic mine water discharged at the +85 m adit portal varies over different periods due to differing conditions such as rainfall and evaporation intensity. Lujiang County, where the alum mine is located, lies between the Yangtze and Huaihe Rivers and is characterized by an uneven intra-annual precipitation distribution with distinct seasonal phases. Consequently, we conducted tests during the wet, normal, and dry seasons (July, October, and December, respectively).

**Figure 7:**
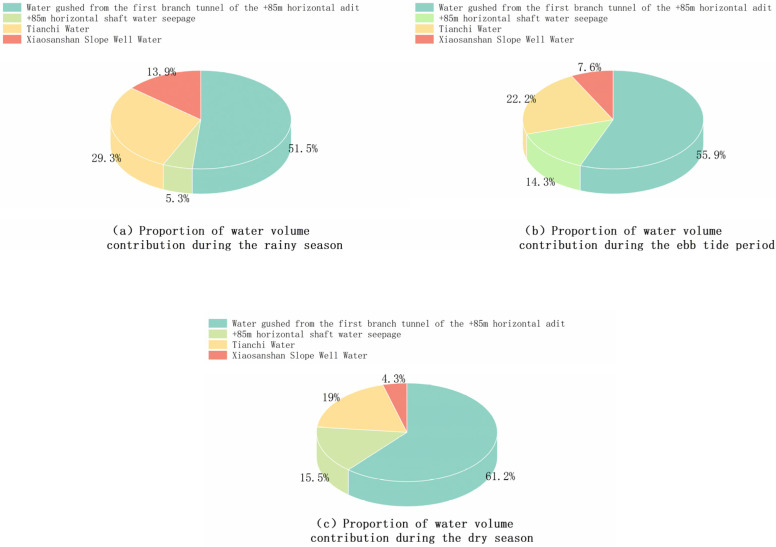
Pie charts of water contribution ratios from different sources over various periods.

The proportional contributions to the mine’s generation of acidic water in the study area are as follows: During the wet season (Figure 7a), water inflow from the First Branch of the +85 m adit contributes 51.44%, seepage within the +85 m adit contributes 5.32%, Tianchi Lake water contributes 29.34%, and the Xiaofanshan Inclined Shaft water contributes 13.90%. During the normal season (Figure 7b), water inflow from the First Branch of the +85 m adit contributes 55.91%, seepage within the +85 m adit contributes 14.31%, Tianchi Lake water contributes 22.18%, and the Xiaofanshan Inclined Shaft water contributes 7.60%. During the dry season (Figure 7c), water inflow from the First Branch of the +85 m adit contributes 61.19%, seepage within the +85 m adit contributes 15.52%, Tianchi Lake water contributes 18.96%, and the Xiaofanshan Inclined Shaft water contributes 4.33%.

### 3.4. Quantification of Contributions of Water from Different Sources to Acidic Mine Drainage

**Figure 8:**
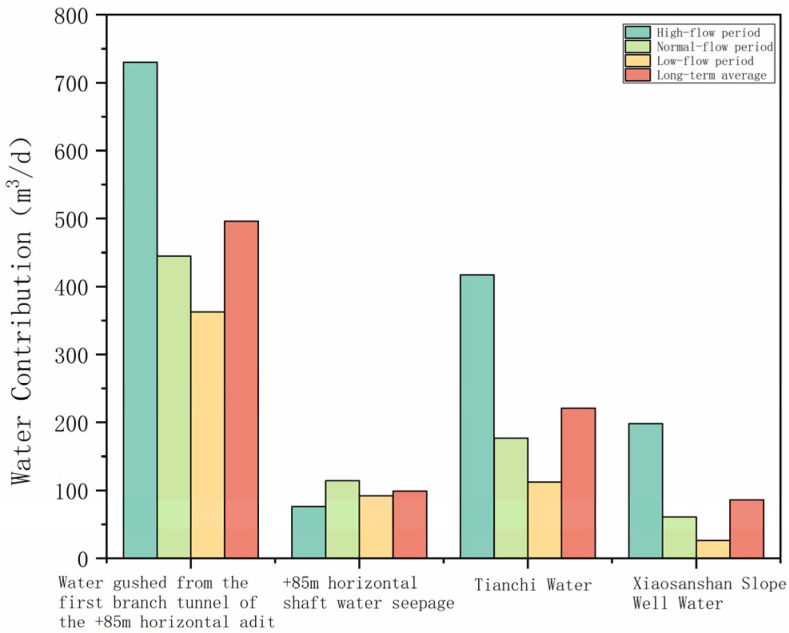
Chart of the contribution of water from various sources to acidic water after remediation.

The contribution of water from various sources to acidic mine drainage in different seasons was determined by integrating the predicted total acidic mine water discharge from the aforementioned groundwater flow model with the proportional contributions of each source calculated using the complete mixing model for the wet, normal, and dry seasons. The detailed results of these calculations are presented in Figure 8.

## 4. Discussion

### 4.1. Analysis of Acidic Water Sources and Recharge Modes Under the Influence of Remediation Disturbance

**Figure 9:**
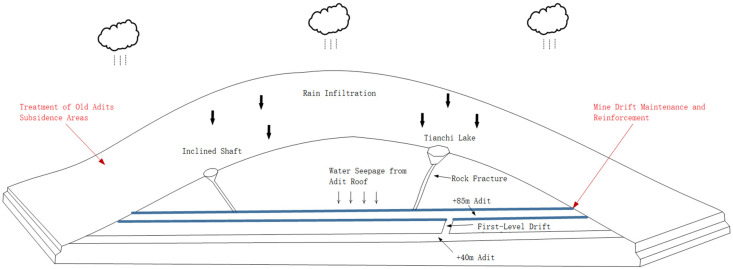
A schematic diagram of the production of acid water via artificial disturbance.

In the alum mine ecological restoration project, the surface engineering components—focusing on the open pit, subsidence area, and mine entrances—underwent site leveling and vegetation restoration. This involved backfilling earthworks according to the existing topography and implementing seepage control through the planting of acid-tolerant plants and microbial treatments. The underground engineering components primarily involved the maintenance and reinforcement of the main tunnel of the +85 m adit to reduce the inflow of acidic water into the mine workings. The specific sources of the acidic wastewater are detailed in Figure 9.

The restoration project effectively restored the surface catchment and groundwater seepage conditions that were altered by long-term alum mining, leading to successful control of the total acidic water volume. However, the current discharge within the adit remains significant. Based on field investigations and geophysical survey data, rainfall infiltration remains the primary source of acidic water in the mining area. The inferred recharge modes are as follows:

(1) Rain in the study area infiltrates through mining-induced voids and rock fractures down to the +40 m adit. It then uses the mining passage between the +40 m adit and the +85 m adit (the First Branch of the +85 m adit) as a flow conduit, ultimately discharging into the +85 m adit and mixing with the acidic water in the main drainage channel.
(2) Rain infiltrates through mining-induced voids and rock fractures to the exterior of the +85 m adit. After reacting with concrete and microbial additives, the acidic water corrodes and compromises the concrete structure, penetrating the concrete support and entering the main drainage channel. Water in this form has a weakly alkaline pH, and while the seepage volume from individual points is small, the extensive and complex network of the +85 m adit, with its considerable total length, makes the collective contribution from this recharge mode non-negligible and even significant.
(3) A deep pond (“Tianchi”) that formed in a subsidence area within the study area receives rainfall via surface runoff. Field analysis indicates that the bottom of Tianchi is connected to the +85 m adit through fractures, providing a direct conduit for rapid recharge to the +85 m adit. This pond contributes a substantial volume of water to the main drainage channel.
(4) In the Xiaofanshan area within the study area, an inclined shaft (the Xiaofanshan Inclined Shaft) remains. Rain enters this shaft as surface runoff. Based on site visits and analysis of mining records, the Xiaofanshan Inclined Shaft is connected to the +85 m adit in the Xiaofanshan area, allowing acidic water to converge into the main drainage channel via this connection.

### 4.2. Analysis of the Acidification Mechanism in Mine Wastewater Influenced by Remediation Measures

As determined through mining data and previous studies, the predominant subsurface rock types in the mining area include alunite, sericite, pyrite, potassium feldspar, kaolinite, acidic tuff, and quartz [19]. The primary chemical constituents of the minerals in this alum mining region are KAl(SO_4_)_2_·12H_2_O (alunogen), SO_3_, FeS_2_ (pyrite), K_2_O, Al_2_O_3_, Na_2_O, CaO, and SiO_2_. The chemical interactions between groundwater and these minerals/rocks—mainly involving KAl(SO_4_)_2_·12H_2_O, SO_3_, CaO, and FeS_2_—are described below.

Alunogen, also known as potassium aluminum sulfate dodecahydrate (KAl(SO_4_)_2_·12H_2_O), reacts with water under normal temperature and pressure conditions, leading to the formation of sulfuric acid. This process acidifies the groundwater flowing through alunogen-bearing zones. More specifically, the mechanism by which potassium aluminum sulfate dodecahydrate generates strong acidity lies in its dissociation in aqueous solutions, releasing sulfate ions and two types of metal cations. The Al^3+^ ion readily undergoes hydrolysis, producing H^+^ ions and Al(OH)_3_.

The gelatinous Al(OH)_3_ formed in the reaction exhibits strong adsorptive capacity, which underpinned the prolonged use of alum as a water purifying agent in China. This colloidal precipitate continuously adsorbs impurities from the groundwater, forming larger aggregates and consequently reducing the aqueous concentration of Al(OH)_3_. This removal drives the equilibrium of the hydrolysis reaction forward, per Le Chatelier’s principle, resulting in the sustained release of H^+^ ions.

The acidic groundwater generated through these reactions subsequently reaches the main drainage channel via multiple recharge pathways, maintaining the consistently low pH observed in the mine effluent.

Fundamentally, the combined presence of H^+^ and SO_4_^2−^ ions effectively creates sulfuric acid, which is the root cause of acidity in the study area’s wastewater. While the concurrent release of K^+^ ions is noted in the ionic reactions, their concentration does not significantly influence the pH of the acidic water and thus warrants no further discussion in this context.

There is a large amount of acidic tuff in the mining area. After the groundwater undergoes the chemical reactions mentioned, sulfuric acid is generated. This sulfuric acid then reacts with CaO present in the tuff, forming calcium sulfate and water. According to previous studies, alunite ores contain a certain amount of SO_3_, which is a strong oxidizing agent. It reacts with water, releasing sulfuric acid. According to alum mining data, the ore in this area contains pyritic alunite ore, which accounts for 56% of the total ore reserves in the mining area. The reaction between pyrite and groundwater cannot be ignored. Pyrite (FeS_2_) is one of the primary sources of acid mine wastewater. Due to prolonged exposure to water, oxygen, and other substances, the sulfur in pyrite is easily oxidized and dissolves in water, leading to a continuous increase in the concentration of SO_4_^2−^ ions in groundwater.

### 4.3. Effects of Amendments on Soil Microbial Community Structure

Based on construction design data and field investigations, the ecological restoration project includes measures that directly affect acidic water quality, namely, tunnel support reinforcement and in situ treatment of acidic mine wastewater in mining cavities. The former involves reactions with acidic wastewater, where acidic water corrodes tunnel support structures and seeps into the main drainage channels. The latter involves injecting microbial agents into the tunnels via surface boreholes to continuously mitigate pollution in the acidic wastewater through microbial reactions.

#### 4.3.1. Reaction Between Acidic Wastewater and Concrete Support in Adits

The cement used in the concrete for tunnel reinforcement primarily consists of C3A, C4AF, C3S, C2S, and free MgO and CaO. After undergoing hydration reactions, these compounds form calcium aluminate hydrate (CAH), calcium silicate hydrate (C-S-H), calcium sulfoaluminate hydrates (AFm and AFt), calcium hydroxide, and other products.

Under alkaline conditions, the cement and its hydration products in the concrete used for tunnel reinforcement remain stable. For example, calcium silicate hydrate (C-S-H), calcium aluminate hydrate (CAH), calcium sulfoaluminate hydrates (AFm and AFt), and calcium hydroxide (CH) are stable at pH 10.4, pH 11.4, pH 10.7, and pH 12.23, respectively [20].

However, under the acidic conditions prevalent at the mine site, hydrogen ions (H^+^) in the groundwater readily react with the hydration products, leading to concrete corrosion [21]. Furthermore, the acidic wastewater in the mining area contains high concentrations of sulfate ions (SO_4_^2−^). These ions penetrate the concrete, react with the hydration products, consume hydroxide ions, and damage the concrete’s structure. The sequence of corrosion by H^+^ and SO_4_^2−^ is as follows.

First, the two corrosive ions react with calcium hydroxide (CH) in a neutralization reaction, reducing the hydroxide ion concentration in the concrete and thereby lowering the pH of the pore solution. However, the stability of various hydration products in the concrete relies on the dissolution of calcium hydroxide (CH). Thus, after prolonged exposure to acidic wastewater, the calcium hydroxide in the concrete is continuously consumed. Once the pH of the pore solution drops below the aforementioned stability thresholds, other hydration products in the cement undergo hydrolysis or react with the two corrosive ions. The corrosion of concrete by H^+^ and SO_4_^2−^ is a prolonged process featuring the following chemical reactions:

(2) $$\begin{array}{c} 3\mathrm{CaO}\cdot 2{\mathrm{SiO}}_{2}\cdot 3H_{2}O+6H^{+}\overset{H_{2}O}{\to }3{\mathrm{Ca}}^{2+}+2({\mathrm{SiO}}_{2}\cdot nH_{2}O)\;+\;6H_{2}O\\ \mathrm{Ca}{\left(\mathrm{OH}\right)}_{2}+2H^{+}\to {\mathrm{Ca}}^{2+}+2H_{2}O\\ C_{3}A\cdot {3\mathrm{CaSO}}_{4}\cdot 32H_{2}O+12H^{+}\to 3{\mathrm{Ca}}^{2+}+{2\mathrm{Al}}^{3+}+{3\mathrm{CaSO}}_{4}+32H_{2}O \end{array}$$

The groundwater in the mining area contains a high concentration of SO_4_^2−^ ions, which react with the concrete in the following way:

(3) $$\begin{array}{c} {\mathrm{Ca}}^{2+}+{\mathrm{SO}}_{4}^{2-}+{2H}_{2}O={\mathrm{CaSO}}_{4}\cdot {2H}_{2}O\\ (3\mathrm{CaO}\cdot {\mathrm{Al}}_{2}O_{3}\cdot {\mathrm{CaSO}}_{4}\cdot {18H}_{2}O)+{2\mathrm{CaSO}}_{4}+{14H}_{2}O\\ \to 3\mathrm{CaO}\cdot {\mathrm{Al}}_{2}O_{3}\cdot {3\mathrm{CaSO}}_{4}\cdot {32H}_{2}O \end{array}$$

As the reactions progress, the acidic groundwater within the study area continuously corrodes the concrete support of the adits, leading to the emergence of numerous water seepage points on the adit ceilings. The water seeping into these adits exhibits weak alkalinity and a high concentration of Ca^2+^, aligning with the reactions described above.

#### 4.3.2. Reaction Between Acidic Wastewater and Microbial Additives

The remediation project included diverting catchment areas and restoring vegetation. These measures significantly reduced the volume of acidic wastewater infiltrating the mine cavities as well as its dissolved oxygen and heavy metal pollution loads. Nevertheless, due to the extensive exposure of alunite and other sulfide minerals to air and water within the cavities and along groundwater paths, oxidation–dissolution reactions mediated by iron/sulfur-oxidizing microorganisms will continue. Therefore, the generation of acidic wastewater is expected to persist in the long term.

Consequently, addressing this persistent problem necessitates an in situ remediation strategy that employs anaerobic microbial processes, with a specific focus on sulfate reduction. This method is highly advantageous, as sulfate-reducing bacteria (SRB) produce net alkalinity to counteract acidity while concurrently facilitating the removal of sulfate and dissolved metal ions via their precipitation as metal sulfides. This dual capability validates its preference as a remediation technique [22].

## 5. Conclusions

Through hydrochemical analysis, we determined that the acidification of acid mine drainage in the study area primarily stems from the hydrolysis of alum (mainly KAl(SO_4_)_2_·12H_2_O) and the oxidation of pyrite (FeS_2_). In response to this issue, remediation measures have triggered two main chemical reactions: (1) neutralization between acidic water and cement in concrete support structures, leading to an increase in pH, and (2) artificially mediated microbial sulfate reduction, which generates hydrogen sulfide and alkalinity, thereby promoting metal precipitation and further acid neutralization.

Under the implemented remediation scenarios, the annual production of acid mine drainage is projected to reach 3.29 × 10^5^ m^3^/year. The first branch tunnel of the +85 m adit constitutes the primary source, accounting for 50–60% of the total discharge, followed by Tianchi Lake, at 20–30%. The contributions from roof seepage in the +85 m adit and water inflow from the inclined shaft are relatively minor and comparable in magnitude.

## Figures and Tables

**Table 1:** Basic information about the sampling point.

Number	Name	Latitude	Longitude	Elevation (m)
S1	+85 m Adit Main Drainage Ditch Water	117.42030323	31.09140902	85
S2	Water Inflow from First Branch, +85 m Adit	117.41940737	31.09107941	85
S3	Roof Seepage Water, +85 m Adit	117.41695046	31.09020659	88
S4	Tianchi Lake Water	117.41484761	31.09231514	178
S5	Xiaofanshan Inclined Shaft Water	117.40213394	31.08465706	113
S6	Water from Pond at Mountain Foot	117.40509510	31.09916417	74
S7	Residential Well Water	117.40841031	31.09820873	69

**Table 2:** Sampling frequency statistics in 2023.

Sample	Flow Rate	pH	Hydrochemistry	δ^2^H & δ^18^O Isotopes	^222^Rn Isotope
S1	12 times	12 times	1 time	1 time	15 times
S2	/	12 times	1 time	1 time	15 times
S3	/	12 times	1 time	1 time	15 times
S4	/	12 times	1 time	1 time	15 times
S5	/	12 times	1 time	1 time	15 times
S6	/	12 times	1 time	1 time	15 times
S7	/	12 times	1 time	1 time	15 times
Note	Monthly	Monthly	Concurrent testing (aligned with the radium isotope measurement period during normal water conditions)		Three phases—high-, normal-, and low-water-level periods—each with five consecutive days of measurement

**Table 3:** Initial value table regarding hydrogeological parameter zoning.

Stratum	Zone	Horizontal Permeability Coefficient (m/d)	Vertical Permeability Coefficient (m/d)	Specific Yield	Elastic Storage Coefficient (m/d)
Layer 1	I	0.5	0.05	0.09	0.0009
	II	1.3	0.13	0.15	0.0015
	III	5	0.5	0.25	0.0025
Layer 2	I	1.3	0.13	0.05	0.0005
	II	1	0.1	0.05	0.0005
	III	0.38	0.04	0.05	0.0005
Layer 3	I	1000	1	0.6	0.0060
	II	0.35	0.05	0.05	0.0005
Layer 4	I	0.27	0.04	0.06	0.0006
Layer 5	I	1000	1	0.6	0.0060
	II	0.29	0.04	0.04	0.0004

**Table 4:** Water quality test results in the study area.

Sample	PH	EC (µs/cm)	TDS (mg/L)	K^+^ (mg/L)	Na^+^ (mg/L)	Ca^2+^ (mg/L)	Mg^2+^ (mg/L)	Fe^2+^ + Fe^3+^ (mg/L)	Mn^2+^ (mg/L)	Al^3+^ (mg/L)	Cu^2+^ (mg/L)	Zn^2+^ (mg/L)	SO_4_^2−^ (mg/L)	Cl^−^ (mg/L)	NO_3_^−^ (mg/L)	NO_2_^−^ (mg/L)	HCO_3_^2−^ (mg/L)	NH_3_-N (mg/L)
S1	3.38	777	388	10	5.85	20.4	4.93	4.12	0.26	19.8	1.76	0.141	44.8	9.79	0.765	0.004	2.98	0.846
S2	2.7	841	423	9.89	3.9	14.6	4.75	7.49	0.26	19.4	1.86	0.086	73.9	10.8	0.867	ND	2.61	0.392
S3	7.18	580	290	26.6	14.7	113	13.2	ND	ND	ND	ND	ND	55.8	3.52	0.393	0.27	124	0.352
S4	4.17	348	174	5.98	5.92	29	7.44	ND	0.21	1.62	0.37	0.062	129	9.88	0.856	ND	13.9	0.138
S5	3.98	615	307	20	5.81	82.7	7.81	ND	0.04	1.76	4.12	ND	95.7	13.9	1.25	ND	6.33	0.114
S6	7.2	1284	642	6.57	7.31	273	64.3	ND	ND	ND	ND	ND	113	15.4	1.28	0.047	44.6	0.136
S7	5.41	147	74	2.53	4.63	13.4	5.06	ND	ND	ND	ND	ND	20.7	4.84	3.07	ND	12.7	ND

ND: Not detected.

## Data Availability

The raw data supporting the conclusions of this article will be made available by the authors on request.
